# The role of dipeptidylpeptidase-4 inhibitors in management of cardiovascular disease in diabetes; focus on linagliptin

**DOI:** 10.1186/s12933-018-0704-1

**Published:** 2018-04-18

**Authors:** Annayya R. Aroor, Camila Manrique-Acevedo, Vincent G. DeMarco

**Affiliations:** 1 Diabetes and Cardiovascular Center, University of Missouri School of Medicine, Columbia, MO USA; 2 Division of Endocrinology and Metabolism, Department of Medicine, University of Missouri-Columbia School of Medicine, One Hospital Drive, Columbia, MO 65212 USA; 3 Research Service, Harry S. Truman Memorial Veterans Hospital, Columbia, MO USA; 4 Department of Medical Pharmacology and Physiology, University of Missouri, Columbia, MO USA

**Keywords:** Vascular dysfunction, Obesity, Insulin resistance, Diastolic dysfunction, Incretin

## Abstract

Multiple population based analyses have demonstrated a high incidence of cardiovascular disease (CVD) and cardiovascular (CV) mortality in subjects with T2DM that reduces life expectancy by as much as 15 years. Importantly, the CV system is particularly sensitive to the metabolic and immune derangements present in obese pre-diabetic and diabetic individuals; consequently, CV dysfunction is often the initial CV derangement to occur and promotes the progression to end organ/tissue damage in T2DM. Specifically, diabetic CVD can manifest as microvascular complications, such as nephropathy, retinopathy, and neuropathy, as well as, macrovascular impairments, including ischemic heart disease, peripheral vascular disease, and cerebrovascular disease. Despite some progress in prevention and treatment of CVD, mainly via blood pressure and dyslipidemia control strategies, the impact of metabolic disease on CV outcomes is still a major challenge and persists in proportion to the epidemics of obesity and diabetes. There is abundant pre-clinical and clinical evidence implicating the DPP-4-incretin axis in CVD. In this regard, linagliptin is a unique DPP-4 inhibitor with both CV and renal safety profiles. Moreover, it exerts beneficial CV effects beyond glycemic control and beyond class effects. Linagliptin is protective for both macrovascular and microvascular complications of diabetes in preclinical models, as well as clinical models. Given the role of endothelial-immune cell interactions as one of the key events in the initiation and progression of CVD, linagliptin modulates these cell–cell interactions by affecting two important pathways involving stimulation of NO signaling and potent inhibition of a key immunoregulatory molecule.

## Background

### Glycemic control, CVD and DPP-4 inhibitors

Overwhelming evidence indicates that CVD risk increases along with increases in glycated hemoglobin (HbA1c). For example, data from the Norfolk study indicated a linear relationship between HbA1c concentrations and CVD and mortality [1]. This analysis revealed that for every percentage point increase in HbA1c above 7%, the relative risk of CVD increases by 20–30% [1]. Surprisingly, this relationship also extended to those below the threshold for controlled T2DM, i.e., HbA1c between 5 and 6.9%. Nonetheless, there is conflicting evidence to support an intensive glucose-lowering regimen in T2DM to reduce major adverse CV events and deaths [2, 3]. Moreover, some conventional diabetes therapies, although effective at glucose control, may actually increase the risk of CVD events, increase hypoglycemic episodes and result in weight gain [4–6]. Newer anti-hyperglycemic drugs, such as DPP-4 inhibitors, GLP-1 agonists and SGLT2 inhibitors, are well tolerated and effective and are increasingly prescribed. These drugs may exert beneficial CV effects beyond glycemic control [7–9], thereby making them attractive strategies as either stand-alone or add-on therapy to conventional glucose lowering medications, such as metformin, sulfonylureas, thiazolidinediones and insulin. The emphasis on their CV safety is becoming an emerging issue. In this regard, large clinical trials have shown either neutral or beneficial effects for DPP-4 inhibitors [10]. Recently, the CV protective effects of different DPP-4 inhibitors have been reviewed [7, 11–14]. It is noteworthy that the DPP-4 inhibitor, linagliptin, has unique kinetics, chemical nature and potent direct effects on the vasculature [15]. In this review we will update recent advances in our understanding of the cellular and molecular mechanisms of CV protection of DPP-4 inhibitors with a focus on linagliptin.

### Cardiovascular protection as a treatment goal in treatment of type 2 diabetes mellitus (T2DM)

Prior to 2008, the US Food and Drug Administration (FDA) approval process for new diabetes therapies was based largely on whether a drug was effective at improving HbA1c and its general safety profile. Following reports that certain antihyperglycemic agents increased CV events [16, 17], the FDA issued new guidelines in 2008 requiring that drug developers perform comprehensive assessment of CV safety on any new diabetes drug to ensure that these therapies do not increase the risk of CV events. Results from three large randomized trials performed over the last 5 years to address the safety and efficacy of DPP-4 inhibitors in T2DM patients at high risk for CV events have been reported [18–24]. The general consensus from these three trials is that DPP-4 inhibitors, *relative to placebo*, do not reduce or increase the risk of the primary composite endpoints of CV death, myocardial infarction, stroke or hospitalization for unstable angina (4 point MACE in TECOS only) when added to standard of care diabetes therapy. Nevertheless, some concerns have been raised for the CV safety profile of saxagliptin which led to slightly more hospitalizations for heart failure (HHF) (3.5 vs 2.8% versus placebo; hazard ratio, 1.27; 95% CI 1.07–1.51; p = 0.007) in the SAVOR-TIMI trial [18] and alogliptin which showed a non-statistically significant risk for HHF in subjects with pre-existing HF in the EXAMINE trial [25]. These adverse events were not detected in the TECOS trial that examined the CV profile of sitagliptin [20]. Whether the disparity between these clinical trials regarding HHF is related to the individual properties of the DPP-4 inhibitors, the subgroups of patients enrolled in the studies, differences in inclusion criteria or other aspects of clinical trial design remains to be determined. However, a recent survey of data from Medicare beneficiaries, older than 65 years of which 55% had baseline CVD, did not demonstrate increased risk of stroke, myocardial infarction or heart failure when comparing DPP-4 inhibitors with sulfonylureas (SU) or thiazolidinediones (TZDs) [26]. Similarly, an analysis of an insurance database in a Korean population reported no increased risk of HHF in DPP-4 inhibitor users (sitagliptin, linagliptin, vildagliptin and saxagliptin) when compared with SU [27]. It is notable that among the DPP-4 inhibitors examined in the Korean study, patients treated with sitagliptin or linagliptin were at lower risk for HF compared to SU therapy. Moreover, risk for MI in patients with pre-existing CVD and stroke were lower in patients treated with DPP-4 compared to SU. Furthermore, a Taiwanese case control study found that DPP-4 use was related to decreased risk of death after an acute myocardial infarction [28]. It is also noteworthy that small clinical studies (< 50 patients) examining the CV effects of DPP-4 inhibitors, including linagliptin, have shown protective effects, including decreases in aortic PWV, improved microvascular function and lower heart failure risk compared to SU [29, 30]. Most of the large trials investigating the CV safety profile of DPP-4 inhibitors (SAVOR-TIMI, TECOS, EXAMINE, and CARMELINA) were designed to compare the DPP-4 inhibitor to placebo; however the ongoing CAROLINA trial was designed to compare the CV effects of linagliptin to *glimeperide as active comparator* and will soon (second half of 2018) provide valuable information regarding the impact of DPP-4 inhibition in diabetic CV outcomes [27, 31, 32]. Lastly, a very recent systematic review and network meta-analysis, designed to evaluate the effects of long-term CV safety of DPP-4 inhibitors (and GLP-1 agonists), showed lower risk of MI compared to SU-based therapies when these drugs are administered for more than 1 year [33].

### CV protection by linagliptin

**Fig. 1:**
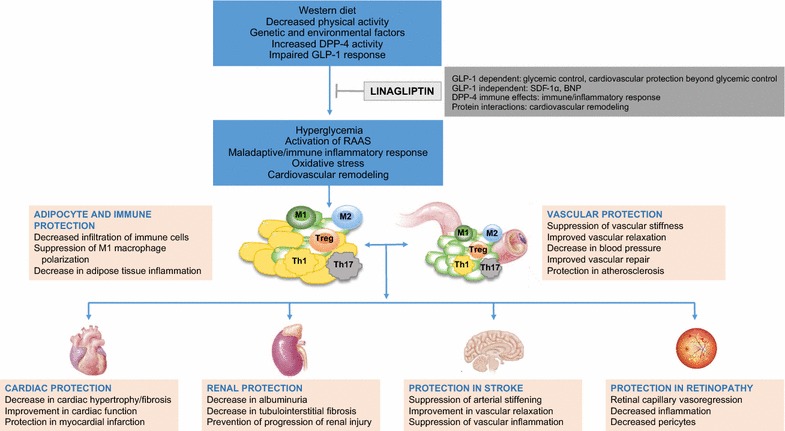
Schematic depicts the cardiovascular protective effects of linagliptin based on pre-clinical and clinical evidence

Both pre-clinical and clinical studies have shown beneficial effects of linagliptin on CV dysfunction associated with obesity and diabetes (Fig. 1). These benefits include improvement in diastolic dysfunction [34, 35], atherosclerosis [36], coronary artery disease (CAD) [37], myocardial infarction [38], hypertension and stroke [39–41], arterial stiffness [29, 42, 43], endothelial dysfunction [30, 37, 44–51], and immune and inflammatory response [35, 52], all of which are depicted in Fig. 1 and reviewed below.

#### Heart failure and diastolic dysfunction

Accumulating evidence indicates that increased circulating DPP-4 activity is associated with poorer CV outcomes in experimental and clinical heart failure models [53]. Further, emerging evidence from preclinical and clinical studies support that DPP-4 inhibitors ameliorate the development and progression of heart failure [27–30, 53]. In this regard, diastolic dysfunction (DD) is one of the early manifestations of CVD in insulin resistant conditions, such as obesity and T2DM and can be identified clinically by echocardiographic findings [54–57]. Moreover, DD is an independent predictor of future CV events, progression to systolic HF and CV mortality and emerging evidence indicates that DD can antedate T2DM and predicts progression of T2DM [58]. Importantly, certain groups are at increased risk of developing DD, including obese children and adolescents [59–61]. Moreover, obese and diabetic premenopausal women are also at heightened risk for CVD when compared to men [54, 55, 62–66]. Significantly, preclinical data with DPP-4 inhibitors have shown promise to improve DD in both males and females [34, 67, 68]. Pre-clinical studies have demonstrated CV protective effects of DPP-4 inhibition in models of genetic and dietary induced obesity, as well as pressure overload [34, 67–69]. We previously tested whether linagliptin reduces pathophysiologic abnormalities in diastolic and vascular endothelial dysfunction in two translationally relevant rodent models of obesity and insulin resistance, the Zucker Obese (ZO) rat [34] and the WD-fed mouse [35, 42]. In one study, male ZO rats were treated for 2 months with linagliptin [34], beginning at 2 months of age when they already display insulin-resistance, DD and mild hypertension [70]. Linagliptin-treated rats exhibited significant improvement in impaired LV diastolic function, as well as endothelial function of gastrocnemius feed arteries, and, somewhat surprisingly, this was associated with a reduction in BP [34]. We extended our investigation of the cardioprotective effects of linagliptin using a dietary murine model of over-nutrition in which 4 week old female mice were fed a high fat-high fructose diet for 4 months (WD-western diet) [35]. Unlike ZO rats that become hypertensive at an early age, 4 months of WD feeding does not induce hypertension in young female mice on a C57Bl/6J background. Our results show that linagliptin exerts robust cardioprotective effects, including the suppression of WD-induced DD, myocardial oxidative stress and inflammation [35]. These promising preclinical findings in translationally relevant models suggest that linagliptin may prevent the onset of DD in insulin resistant states caused by overnutrition, as well as improve DD in the setting of established insulin resistance, obesity and T2DM when there is a pre-existing cardiac relaxation abnormality.

#### Atherosclerosis, coronary artery disease (CAD) and myocardial infarction

The incidence of atherosclerosis and coronary artery disease (CAD) in patients with T2DM is greatly increased compared to individuals without diabetes [71]. Moreover, in the presence of CAD, T2DM subjects have worse clinical outcomes when compared with patients without diabetes [10]. Atherosclerosis accounts for half of all deaths in western countries and is increasing globally [14, 72–74]. DPP-4 inhibitor therapy has been shown to reduce the risk for atherosclerosis and CAD through both glycemic control and direct effects on the atherosclerotic process, including atherosclerosis or plaque stability, and this topic has been addressed in recent reviews [11, 14]. Results have been mixed with respect to the role of DPP-4 inhibition on improvement in cardiac function and remodeling in experimental models of myocardial infarction [38, 53, 75–80]. In this regard, linagliptin has been shown to significantly reduce infarct size and fibrosis after ischemia/reperfusion (I/R) injury in a rat model [38] in association with a significant increase in plasma GLP-1 levels [38]. These salutary effects were not accompanied by improved cardiac function. Despite these mixed results in preclinical settings, it has been reported that DPP-4 inhibitors improve DD or long term survival in T2DM patients after acute myocardial infarction [28, 33, 81]. Moreover, this improvement occurred in both sexes showing CV protection regardless of sex [28].

#### Hypertension and stroke

Hypertension is twice as prevalent in individuals with T2DM compared to non-diabetic individuals [82]. Blood pressure (BP) responses to DPP-4 inhibitor therapy in humans are either neutral [83, 84] or modestly reduced [85–87]. In addition, linagliptin tended to further improve BP in a rat model of renovascular hypertension when administered along with the angiotensin receptor blocker (ARB), telmisartan [41], thereby suggesting that a combination of ARB/DPP-4i could be an additional option for the management of hypertension in T2DM patients.

Recent studies show a lower incidence of non-fatal stroke events in patients treated with linagliptin compared to glimepiride, thereby accounting for significantly fewer major CVD events for linagliptin treated patients compared to those receiving glimepiride [39]. The possibility that incretin enhancer therapy could be neuroprotective is likely given that GLP-1 receptors are expressed in neurons from rodents and humans [88, 89] and that native GLP-1 and GLP-1 analogs readily cross the blood brain barrier [90, 91]. Previous studies demonstrate that exendin-4, a GLP-1 receptor (GLP-1r) agonist, abrogates the severity of stroke in diabetic and non-diabetic rodent models [92–94]. Linagliptin has also been tested for its efficacy in reducing complications from stroke utilizing middle-aged non-diabetic and diabetic mice subjected to middle cerebral artery occlusion [40]. In addition to reducing plasma DPP-4 activity, linagliptin increased plasma GLP-1 levels. This was associated with a significant increase in the number of surviving cortical neurons, despite no reduction in brain infarct size, in both non-diabetic and obese diabetic mice. GLP-1 mediated modulation of matrix metalloproteinases appears to be one of the important mechanisms contributing to vasculoprotective effects of linagliptin [93, 95–97]. There is also evidence indicating that linagliptin can restore impaired cerebrovascular structure and function [98–100]. Linagliptin has also been shown to ameliorate impaired cognitive function and brain atrophy induced by transient cerebral ischemia in diabetic db/db mice [101]. Thus, the cerebro-protective effects of DPP-4 inhibition may be another consideration for treatment of T2DM patients at risk for development of cerebrovascular disease or cognopathy.

#### Arterial stiffness, endothelial dysfunction and CVD

Arterial stiffness is an independent risk factor for CVD, including hypertension, heart failure with preserved ejection fraction, chronic kidney disease and stroke [102, 103]. Arterial stiffness is more prevalent in older individuals [104] and occurs naturally with aging as a consequence of fragmentation and degradation of elastin in the wall of the aorta and its replacement with much stiffer collagen fibers [105]. Nonetheless, arterial stiffness can develop in younger individuals in the setting of insulin resistance, obesity and T2DM and evidence indicates that obese, insulin resistant and diabetic women are more prone to develop vascular stiffness than men [106, 107]. The cellular and molecular mechanisms underlying vascular stiffening comprises endothelial stiffness/dysfunction, increased vascular tone, remodeling of extracellular matrix and dysfunction of adventitial and perivascular adipose tissue [108–110]. Recent reports from our laboratory indicate that therapies targeting vascular stiffness could potentially improve CV outcomes in insulin resistance models [108, 111, 112]. We also reported that administration of linagliptin prevented the development of WD-induced aortic stiffness by an NO-dependent mechanism [42]. Moreover, recent clinical studies reported that linagliptin decreased aortic PWV in subjects with T2DM [29, 43]. These clinical results are consistent with our preclinical study of prediabetic mice fed an obesogenic diet in which linagliptin prevented arterial stiffening and vascular remodeling [42].

The vascular endothelium serves as interface between blood and surrounding tissue components and regulates normal vascular functions including control of vascular tone, extracellular matrix remodeling, coagulation, leukocyte trafficking and permeability, and immune and inflammatory responses [108, 113, 114]. Endothelial dysfunction is caused by both insulin resistance and hyperglycemia in diabetes mellitus and is associated with both development of macrovascular and microvascular complications of T2DM [115, 116]. In addition to protection of macrovascular complications of T2DM by DPP-4 inhibitors, including linagliptin [29, 43, 84, 117], emerging evidence from clinical studies conducted on small numbers of patients with T2DM reported improvement in microvascular function by linagliptin [30, 49–51]. Endothelial dysfunction is usually indicated by decreased bioavailable NO in response to acetylcholine/insulin mediated vascular relaxation [34, 111] or impaired flow mediated vasodilation [29, 43, 84, 117], is strongly associated with insulin resistance and hyperglycemia in diabetes mellitus. In this regard, linagliptin has potent nitric oxide enhancing effects on vascular function [34, 118, 119].

### Renoprotection

Development of kidney disease is one of the major sequelae of T2DM with approximately 50% of diabetic individuals progressing to chronic kidney disease (CKD) [120, 121]. Moreover, CKD is associated with development of CVD including arterial stiffness, hypertension and cardiac dysfunction [122]. Compared to other tissues, the kidneys express the highest level of DPP-4 and it is likely that the presence of DPP-4 in the glomerular endothelium and proximal renal tubules contributes importantly to sodium retention, tubular injury and glomerular injury. We and others have shown renoprotective effects of DPP-4 inhibitors, including linagliptin, in preclinical studies [48, 123–128]. Similarly, the potential beneficial effects of DPP-4 inhibitors, including linagliptin, in preventing and treating progression of kidney disease in patients with T2DM is supported by retrospective analyses of clinical trials [49, 129]. The ongoing Cardiovascular and Renal Microvascular Outcome study with Linagliptin in patients with T2DM (CARMELINA), which is powered to evaluate kidney outcomes and renoprotective effects of this inhibitor, should begin to fill in a gap in our knowledge regarding the efficacy of DPP-4 inhibitors in T2DM patients with CKD with our without CVD [130].

With regard to treating diabetic nephropathy, the pharmacokinetics and pharmacodynamics of linagliptin make it an especially attractive drug for several reasons. First, unlike other DPP-4 inhibitors that are excreted largely in urine, linagliptin is mainly eliminated by a biliary route [131] and therefore does not require dose adjustment in patients with kidney disease [132, 133]. Additionally, compared to other DPP-4 inhibitors, linagliptin can penetrate deeply into renal tissue and therefore has the largest volume of distribution, and has the highest binding affinity for DPP-4 protein that is richly present in kidney [134–137]. Microvascular dysfunction is one of the major factors contributing to progression of diabetic nephropathy and DPP-4 inhibitors have been shown to exert microvascular protection in pre-clinical studies. Importantly, data from prospective clinical trials is beginning to emerge [44, 45, 138]. In this regard, linagliptin-mediated CV effects can occur both in response to better glycemic control, as well as by mechanisms independent of glycemic control in animal models of pre-diabetes [35, 42, 44–48]. Emerging evidence from clinical studies conducted on small numbers of patients with T2DM reported improvement in microvascular function by linagliptin [30, 49–51]. Impairment of NO signaling is one of the pathways that contributes to CVD and linagliptin has potent nitric oxide enhancing effects on vascular function [34, 118, 119]. The nephroprotective effects of DPP-4 inhibitors, including linagliptin, and their potential underlying mechanisms have been the focus of several very extensive and recent reviews [121, 129, 139].

### Cellular and molecular mechanisms

#### GLP-1-dependent and -independent effects

**Fig. 2:**
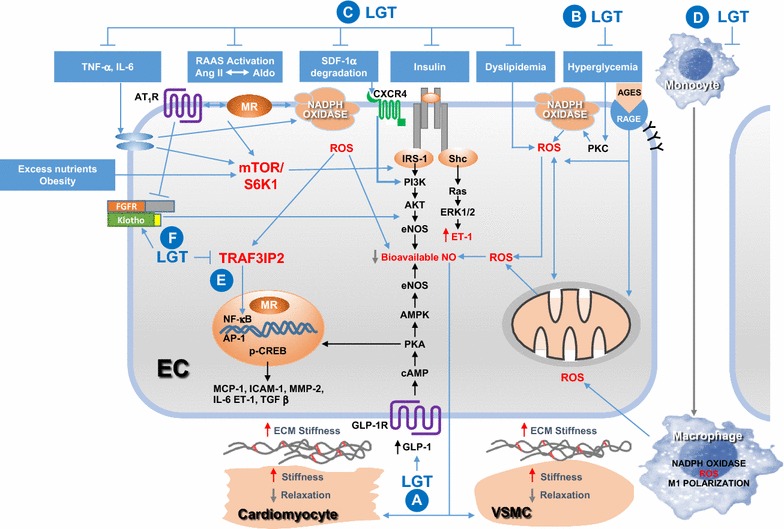
Cellular and molecular mechanisms of linagliptin mediated cardiovascular protection. The schematic depicts deleterious effects of excess nutrient consumption/obesity in the development of cardiometabolic syndrome and T2DM leading to vascular injury, stiffening and cardiovascular dysfunction. Circles with letters A through F indicate targets of LGT-mediated CV protection due to LGT modulation of key pathophysiological events. **a** The classical effects of LGT through inhibition of DPP-4 leading to increased levels of GLP-1 incretin results in GLP-1-mediated cell signaling cascade implicated in improved endothelial function and endothelial regulation of other vascular cells and cardiomyocytes. **b** In addition to cell-specific effects, the glycemic control by GLP-1 incretin signaling contributes to CV health by suppressing deleterious effects of hyperglycemia directly and amelioration of CV injury by AGE/RAGE signaling. The mechanisms largely involve oxidative stress mediated by both NADPH oxidase-dependent, as well as, mitochondrial generated oxidative stress. The enhanced oxidative stress, in turn, contributes to impairment in two key cellular events comprising decrease in bioavailable NO and upregulation of a proinflammatory response. **c** The GLP-1-independent mechanisms of LGT include modulation of cytokine imbalance, RAAS activation, potentiation of SDF-1α signaling to NO, improvement in insulin signaling and suppression of dyslipidemic effects on vasculature. **d** As DPP-4 is expressed in immune cells and mounts a pro-inflammatory response through macrophage and lymphocyte polarization, LGT is an effective suppressor of maladaptive immune/inflammatory response. **e** The recent studies demonstrating LGT-mediated decrease in the levels of TRAF3IP2, which is a key modulator of inflammatory and pro-fibrotic responses in the WD-fed heart, provides novel insight into the effects of LGT in improving CV dysfunction in obesity cardiomyopathy. **f** Recent studies indicate that linagliptin prevents WD-induced deficiency of the anti-aging protein, klotho, in the aorta of WD fed female mice and that the salutary effects of linagliptin on klotho involve increased bioavailable NO. Taken together, the multiple cellular mechanisms of LGT may be contributing to the beneficial effects of LGT observed in pre-clinical models of obesity and diabetes, as well as, small clinical trials showing CV protection in stroke, myocardial infarction and nephropathy. *AC* adenylate cyclase, *AGE* advanced glycation end products, *AP*-*1* activator protein-1, *AT1R* angiotensin type 1 receptor, *AMPK* AMP-activated protein kinase, *AKT* protein kinase-B, *Ang II* angiotensin 2, *Aldo* aldosterone, *p*-*CREB* phospho-cyclic AMP response element binding, *EC* endothelial cell, *ET*-*1* endothelin-1, *ERK* extracellur signal-regulated kinase, *eNOS* endothelial nitric oxide synthase, *FGFR* fibroblast growth factor receptor, *GLP*-*1* glucagon-like peptide-1, *GLP*-*1R* GLP-1 receptor, *HO*-*1* hemoxygenase-1, *IL*-*6* interleukin-6, *LGT* linagliptin, *MCP*-*1* monocyte chemoattractant protein-1, *MMP*-*2* matrix metalloproteinase-2, *MR* mineralocorticoid receptor, *NF*-*κB* nuclear factor-kappa B, *PI3K* phosphoinositide 3-kinase, *RAGE* receptor for AGE, *ROS* reactive oxygen species, *S6K1* ribosomal protein S6 kinase 1, *TGF*-*β* transforming growth factor-1, *TRAF3IP2* TRAF3 interacting protein 2, *mTOR* mechanistic target of rapamycin

The beneficial effects of DPP-4 inhibitors on the CV system (Fig. 1) may occur through glycemic control, as well as mechanisms beyond glycemic control. Linagliptin mediated increase in GLP-1 levels through its classical effect to inhibit DPP-4 activity may partly account for improvement in vascular function and associated improvement in cardiac function (Fig. 2a–c). However, GLP-1-independent mechanisms beyond glycemic control, may also significantly account CV protection by linagliptin [11, 12, 14]. These mechanisms mainly include linagliptin suppression of inappropriate RAAS activation and maladaptive immune and inflammatory response [140] (Fig. 2c, d). The improvement in nitric oxide signaling contributes significantly to suppression of inflammation and improvement of insulin signaling and vascular function, thereby inhibiting the development of atherosclerotic vascular disease [141]. Recent studies also show modulatory effects of linagliptin on TRAF3IP2 signaling (Fig. 2e) and klotho/FGF23 signaling (Fig. 2f) [35, 42].

Pre-clinical studies demonstrate cardioprotective effects of GLP-1 agonists and favor the view that inhibition of DPP-4 resulting in increased levels of GLP-1 is one of the major pathways for CV protection by DPP-4 inhibitors [11, 12]. The cardioprotection by GLP-1 includes improvement in coronary blood flow [142, 143], decreases in cardiomyocyte apoptosis [144], and reduction in infarct size [97, 145]. GLP-1 signaling improves CV function by modulating various signaling pathways, including NO/cGMP, PKA and Akt [11, 146, 147]. A recent study showed GLP-1 mediated suppression of platelet activation thereby demonstrating one more mechanism for the anti-atherosclerotic effects of DPP-4 inhibitors [148].

In addition to GLP-1 mediated effects, DPP-4 inhibitors may have CV protection through GLP-1-independent mechanisms, including inhibition of degradation of other DPP-4 substrates such as gastric inhibitory peptide (GIP) and SDF-1α [12, 14, 31]. Moreover, direct effects of DPP-4, independent of its substrate effects, have also been reported, which in turn may account for CV effects of DPP-4 inhibitors [11, 12, 14].

#### Immune and inflammatory mechanisms

The role of maladaptive innate and adaptive immune and inflammatory responses contributing to CV stiffening and fibrosis is evidenced by changes in the polarization status of T lymphocytes and macrophages [110]. Macrophage polarization with predominant M1 pro-inflammatory response in visceral adipose tissue and perivascular adipose tissue results in increased pro-inflammatory cytokines in plasma and in the vascular wall [149–154]. In addition to macrophages, T cell activation and dysregulation of T-cell polarization can also contribute to CV dysfunction. Moreover, T helper (Th) 1 cells not only induce a pro-inflammatory response, but also promote infiltration of M1 macrophages into adipose and CV tissues [150]. Th17 cells are another subset of CD4^+^ cells that secrete IL-17 which promotes CV injury in obesity, diabetes and hypertension [155]. CD4^+^ CD25^+^ T regulatory cells (Tregs) are a subpopulation of T-cells [156] that mediate anti-inflammatory effects by suppressing pro-inflammatory T-cell responses and promoting M2 macrophage polarization. IL-10 secreted by Tregs inhibits NADPH oxidase mediated oxidative stress thereby contributing to suppression of CV inflammation, improvement of cardiac function and lowering of blood pressure [157, 158].

DPP-4, also known as CD26, is a T-cell surface marker that is widely expressed in immune cells [159] and cleaves numerous chemokines and peptide hormones regulating the immune system, including CCL5, CXCL12, CCL22 and MIP-1α [52, 160–162]. Therefore, DPP-4 inhibitor therapy may be beneficial in suppressing maladaptive innate and adaptive immunity [163] by regulating T cell activation and macrophage polarization in adipose and CV tissue [156, 164–167]. In addition, DPP-4 expressed in dendritic cell/macrophages contributes to potentiating inflammation of adipose tissue in obesity [168]. DPP-4 is also characterized as an adipokine and regulates insulin sensitivity in adipose tissue and other insulin sensitive tissues and organs [169, 170]. DPP-4 is also expressed in F4/80^+^ M1 macrophages [52]. Recent studies show that linagliptin not only reduces migration of M1 polarized macrophages, but also induces M2 dominant macrophage phenotype within white adipose tissue as well as liver that resulted in suppression of inflammation and insulin resistance. Moreover, it decreased the expression of macrophage inflammatory protein-1α (MIP-1α) which is a chemokine, as well as, a DPP-4 substrate. In this regard, linagliptin was not effective in suppressing M1 polarization and insulin resistance in MIP-1α knock down mice suggesting that MIP-1α is a potential mediator contributing to immunoprotective effects of linagliptin [52].

In addition to the direct effect of DPP-4 on immune and inflammatory response, DPP-4 also suppresses RAAS-mediated immune responses [35, 156, 171]. Inappropriate activation of RAAS modulates activation of T-lymphocytes and macrophages [150, 151, 156, 172], thereby contributing to CV dysfunction in obesity and T2DM [150, 173]. In this regard, recent studies showed attenuation of Ang-2 induced cardiac fibrosis by alteration of AT1/AT2 receptor expression and ACE activity in rat hearts [174]. We recently reported increased myocardial expression of AT1r and MR in WD fed female mice; linagliptin suppressed elevated RAAS receptor expression with concomitant suppression of cardiac fibrosis and immune and inflammatory response [35].

#### Nitric oxide signaling

Nitric oxide is vital to CV homeostasis because it is a key regulator of, among other things, vascular function and remodeling and immune and inflammatory responses [175]. Nitric oxide regulates vascular flow, tone, monocyte activation and platelet aggregation, thereby modulating blood pressure, cardiac function, thrombosis, and atherosclerosis [175, 176]. Impairment of NO signaling is associated with most CVDs and is the hallmark of obesity and T2DM [175–177]. Therefore, strategies that modulate NO signaling by way of enhancing endogenous NO signaling or its downstream signaling intermediates, or through delivery of NO precursors, are likely to have salutary effects in the CV tissue. In this regard, recent studies suggest that DPP-4 inhibitors exert CV protection by increasing bioavailability of NO in the vasculature [34, 42, 118, 178]. This occurs by both GLP-1-dependent and -independent mechanisms. *Compared to other DPP*-*4 inhibitors,* linagliptin exerts more potent vasodilatory effects in *aortic rings and these effects are* mediated by activation of the eNOS/Akt, NO/cGMP pathway [118, 178]. Consistent with these mechanistic studies performed ex vivo, previous reports demonstrate that long term administration of linagliptin improves vascular function and NO signaling in both genetic and dietary models of obesity, in both the presence and absence of BP change [34, 42]. Thus, the preclinical evidence suggests that the NO enhancing effects of linagliptin could translate into improved CV outcomes in patients with T2DM.

#### FGF23/klotho signaling

Klotho is an anti-aging protein that has received much attention for its role as an aging suppression gene [179]. Klotho is expressed in high amounts in kidney and lesser amounts in parathyroid cells, adipocytes, brain and vascular endothelial cells [180–182]. The cardioprotective effects of klotho have been recently reported [183]. Among the downstream targets of klotho, Sirt1 and AMPK are considered to be CV protective molecules [184]. Importantly, aging is associated with a decrease in circulating klotho levels [185]. Emerging evidence indicates klotho deficiency may be a major contributor to not only, age-related aortic stiffening, but also obesity-associated aortic stiffness. In this regard, mice with a genetic deficiency in the klotho gene develop premature aortic stiffness that is associated with increased collagen deposition and reduced elastin in the medial layer of the aorta [186]. Moreover, klotho deficient mice had elevated circulating aldosterone concentrations and mineralocorticoid receptor blockade prevented the aortic stiffening and remodeling associated with klotho deficiency [186]. Moreover, klotho levels are decreased by Ang-2 [187] and one of the downstream effects of klotho is regulation of NO signaling (Fig. 2c, f) [188].

Emerging evidence suggests a new mechanism to explain the vasculo-protective effects of linagliptin (and perhaps other DPP-4 inhibitors) that involves modulation of aging pathways. We recently reported that long term consumption of a WD induces aortic stiffness in female mice and this was prevented with linagliptin [42]. We also determined that WD induced a deficiency in klotho protein expression in the aorta that was prevented with linagliptin administration [42]. To our knowledge, this was the first study suggesting that the vasculo-protective effects of linagliptin involve modulation of klotho signaling. In addition, a more recent study showed that amelioration of progression of premature aging in klotho knock out mice by linagliptin and these salutary effects were associated with increased bioavailable NO in the cerebral vasculature [188]. Angiotensin II decreases klotho levels and linagliptin improves Ang II signaling, including downregulation of AT1R [35]. Therefore, the klotho-mediated beneficial effects of linagliptin may be accounted for by both modulation of Ang II signaling upstream of klotho (Fig. 2c) and NO signaling downstream of klotho (Fig. 2f).

#### TRAF3IP2 (TRAF3 interacting protein2)

TRAF3IP2 is a key regulator of the immune and inflammatory response and exerts multiple effects to promote CV stiffening, inflammation, and fibrosis that contribute to cardiac dysfunction and vascular inflammation. TRAF3IP2 signaling is a convergent point in regulation of a pro-fibrotic response. The upstream regulators of TRAF3IP2 include oxidative stress, RAAS activation and cytokines, including IL-17, whereas the downstream targets are transcriptional factors, such as NF-κB and AP-1 and cell signaling pathways such as p38-MAPK and the crosslinking enzyme, lysyl oxidase [189] (Fig. 2e). Induction of TRAF3IP2 in cardiac fibroblasts in response to either Ang-2 [190] or aldosterone [191] promotes a fibrotic response. We recently showed upregulation of TRAF3IP2 in heart tissue from WD-fed female mice and linagliptin administration significantly suppressed this induction. We further demonstrated that the transcription factors and kinase signaling pathway regulated by TRAF3IP2 are also upregulated by WD feeding and this was associated with maladaptive cardiac immune and inflammatory response, as well as fibrosis [35]. Supporting these in vivo observations, our in vitro studies using isolated cardiac fibroblasts also demonstrated that linagliptin inhibits aldosterone-induced TRAF3IP2 expression, oxidative stress, inflammatory cytokine expression, and cardiac fibroblast activation and migration. Collectively, these findings suggest that one of the mechanisms by which linagliptin suppresses maladaptive immune and inflammatory response in CV tissue is through modulation of immune regulatory molecules, such as TRAF3IP2.

#### SDF-1 (stromal cell-derived factor-1)/CXCR4 signaling

The tissue repair process in response to CV injury is regulated by multiple factors including recruitment of progenitor/stem cells [192, 193]. Diabetes is characterized by a deficiency or dysfunction in circulating progenitor/stem cells which predicts future CV events, poor macro- and microvascular outcomes and death [194–196]. In this regard, SDF-1α is a CXC chemokine and ligand for the CXCR4 receptor, in addition to being a substrate of DPP-4. SDF-1α is a potent chemoattractant for various stem cells involved in tissue repair and regeneration, including among others, endothelial progenitor cells, endogenous cardiac stem cells, bone marrow stem cells, mesenchymal stem cells, and T-lymphocytes [192]. Tissue injury can induce local endothelial cells to secrete SDF-1α which mediates adherence of circulating stem cells to the endothelium. In the setting of diabetes, SDF-1α may be rapidly degraded by local and circulating DPP-4 activity which may limit mobilization and attachment of stem cells to injured tissue. As there is no effective therapy to treat CV injury and fibrosis associated with T2DM, DPP-4 inhibitor-mediated targeting of SDF-1α, to prevent its degradation and enhance stem cell mobilization from bone marrow and recruitment to peripheral tissue, may be an attractive strategy to enhance CV tissue repair [35, 38, 197–199]. It should be noted that a recent review article has proffered the notion that the potentiation of SDF-1α by DPP-4 inhibitor therapy may exacerbate rather than resolve tissue inflammation and fibrosis, thus neutralizing the benefits of potentiating GLP-1 signaling [200]. This idea has sparked debate [201]. In this regard, DPP-4 inhibitors have not been shown to have CV adverse effects for most of these inhibitors and the adverse effects of saxagliptin may be related to a subset of patients with pre-existing cardiac disease [20, 26, 27, 201]. Moreover, linagliptin has been shown to increase circulating SDF-1α, as well as putative vascular regenerative and anti-inflammatory cells in patients with T2DM, independently of its effects on glycemia [201, 202]. Other studies provide compelling evidence that linagliptin-induced increases in local and circulating levels of SDF-1α are associated with tissue repair. For example, linagliptin and the novel DPP-4 inhibitor, BI 14361 have been shown to significantly reduce infarct size and myocardial fibrosis in an experimental model of myocardial ischemia–reperfusion injury in Wistar rats [38]. Improvement in tissue remodeling in treated rats was associated with accumulation of cells positive for SDF-1α, CXCR-4 and CD34 within and around the infarcted area. This study supports the notion that SDF-1α is upregulated during myocardial ischemia and contributes to mobilization of bone marrow derived stem cells to the ischemic heart. In another study, we reported that linagliptin treatment increased glomerular and kidney tubular expression of SDF-1α, as well as, circulating SDF-1α levels and ameliorated kidney injury in rat model of diabetic nephropathy [48]. Importantly, SDF-1α has been shown to cause eNOS activation in endothelial cells and preserve microvascular integrity [193] and this is depicted in Fig. 2c.

### Linagliptin combination with an SGLT2 inhibitor

The first line therapy for management of glycemia for T2DM throughout the world is metformin [203]; however, over time, the effectiveness of metformin monotherapy in achieving target HbA1c diminishes in a majority of patients [204]. This lack of adequate glycemic control necessitates the use of combinations of one or more additional anti-hyperglycemia agents to achieve target HbA1c. However, interest in the need for combination therapy as an initial treatment strategy is also increasing [205–207]. Recent studies indicate that DPP-4 inhibitors, including linagliptin, could potentially complement the effects of other CV protective agents such as metformin [208], angiotensin type 2 receptor blockers (ARB) [41, 209], statins, and SGLT2 inhibitors [210–212]. Importantly, there is little evidence of undesirable drug interactions between linagliptin and metformin, ARBs, statins and SGLT2 inhibitors [5, 213]. The increased risk of angioedema with combination DPP-4 and ACE inhibitor therapy is one potential limitation [214], however, this issue may be managed by consideration of replacing an ACE inhibitor with an ARB [128, 215].

Recent studies have shown that combination therapy of DPP-4 and SGLT2 inhibitors, either as an initial combination or stepwise addition, results in improvement in glycemic control [216–221]. In contrast to other DPP-4 inhibitors, linagliptin does not require dose adjustment in patients with renal insufficiency given that it is not excreted by the kidneys [15]. Indeed, the evidence so far indicates that once daily single pill combination of linagliptin and the SGLT-2 inhibitor, empagliflozin, results in clinically meaningful and sustained reductions in HbA1c, fasting glucose, body weight and blood pressure [220, 222]. Other combinations of SGLT2 and DPP-4 inhibitors seem similarly promising [220]. The underlying mechanisms for these beneficial effects appears to be the convergence of complementary signaling pathways and physiological effects (e.g., reduced glucotoxicity, weight loss and BP reduction for SGLT2 inhibitor and reduced glucose-dependent glucagon secretion and anti-inflammatory effects for DPP-4 inhibitor) for beneficial effects on CV health or suppression of microvascular complications [220, 223].

## Conclusions

Linagliptin is protective for both macrovascular and microvascular complications of diabetes in preclinical models, as well as clinical models. Linagliptin exerts beneficial CV effects through glycemic control, as well as effects beyond glycemic control and beyond class effects. Linagliptin modulation of endothelial and immune cell responses appear to be key mechanisms for ameliorating the progression of CVD.

## Abbreviations

DPP-4: dipeptidylpeptidase-4
GLP-1: glucagon-like peptide-1
GIP: glucose-dependent insulinotropic peptide
SU: sulfonylureas
TZD: thiazolidinedione
SDF-1α: stromal-cell-derived factor-1α
SGLT2: sodium-glucose co-transporter 2
ARB: angiotensin type 2 receptor blocker
ACE: angiotensin converting enzyme
TRAF3IP2: TRAF3 interacting protein2
MIP-1α: macrophage inflammatory protein-1α
Tregs: T regulatory cells
SAVOR-TIMI: saxagliptin assessment of vascular outcomes recorded in patients with diabetes mellitus (SAVOR)–thrombolysis in myocardial infarction (TIMI)
TECOS: trial evaluating cardiovascular outcomes with sitagliptin
EXAMINE: examination of cardiovascular outcomes with alogliptin versus standard of care
